# Culturally Meaningful End-of-Life Care for a Patient With Cancer of Unknown Primary, and Severe Motor and Intellectual Disabilities: A Case Report

**DOI:** 10.7759/cureus.110869

**Published:** 2026-06-15

**Authors:** Daiki Kogetsu, Yuki Kimoto, Yasutoshi Yamamoto, Katsuji Tanaka

**Affiliations:** 1 Department of Nursing, Nishinomiya-Sunago Medical and Welfare Center for Children with Severe Motor and Intellectual Disabilities, Nishinomiya, JPN; 2 Department of Pediatrics, Nishinomiya-Sunago Medical and Welfare Center for Children with Severe Motor and Intellectual Disabilities, Nishinomiya, JPN

**Keywords:** cancer of unknown primary (cup), ethical decision-making, japanese geriatrics, multidisciplinary team (mdt), palliative and end-of-life care, severe motor and intellectual disabilities, surrogate decision-making

## Abstract

Geriatric and palliative care are becoming increasingly complex, particularly for patients with severe motor and intellectual disabilities (SMID). Cancer of unknown primary origin (CUP) is an aggressive malignancy that is often diagnosed at an advanced stage and requires individualized decision-making and careful integration of patient values into care planning. We report the case of a 68-year-old man with SMID who was diagnosed with peritoneal carcinomatosis of unknown primary origin, accompanied by pulmonary and splenic metastases and peritoneal implants. Because he was unable to express his preferences, repeated discussions with his legal guardian, his aunt, led to a consensus to prioritize comfort-focused care over invasive diagnostics or disease-directed therapy. A culturally meaningful intervention, a music concert featuring favorite songs, was organized within the care facility. The patient exhibited visible emotional engagement that facilitated reconnection with distant relatives. He died peacefully one week later without invasive medical interventions. This case underscores the importance of nonverbal cues, cultural sensitivity, and interdisciplinary collaboration in end-of-life decision-making for patients with profound disabilities. This further demonstrates how personalized non-medical interventions can enhance dignity, emotional comfort, and family cohesion at the end of life.

## Introduction

Japan is experiencing one of the most rapid demographic shifts toward a super-aging society, resulting in increasing demand for sophisticated geriatric and palliative care frameworks [1]. Care of patients with severe motor and intellectual disabilities (SMID) presents unique clinical, ethical, and logistical challenges. SMID is a clinical category used in Japan to describe individuals who are chair-ridden or bedridden and have an intelligence quotient (IQ) of ≤35, as originally defined by Oshima [2]. People with intellectual disabilities often have a limited capacity to articulate symptoms or preferences and may experience heightened vulnerability during hospitalization, including anxiety, sensory overload, and difficulty adapting to unfamiliar environments [3-5]. Therefore, maintaining continuity in the setting and providing individualized person-centered care are essential components of high-quality end-of-life care for this population [6].

Cancers of unknown primary origin (CUPs) represent a heterogeneous group of metastatic malignancies characterized by diagnostic uncertainty, rapid progression, and poor prognosis [7-9]. Previous epidemiological reviews have demonstrated that CUP has a wide survival range, with a median survival of six to seven months in unfavorable‑risk subsets and 12-36 months in favorable‑risk subsets [9]. When CUP occurs in patients with SMID, care complexity increases substantially. Invasive diagnostic procedures may be impractical or ethically inappropriate, and shared decision-making is often constrained by patient communication limitations. As a result, surrogate decision-makers and multidisciplinary teams (MDT) must rely on long-standing behavioral cues, established routines, and knowledge of the patient’s values to guide care planning.

In Japan, palliative care is shaped by several structural and cultural factors that differ from those in Western systems. Advance Care Planning (ACP) has become widespread only recently, and surrogate decisions are often made without formal documentation of patient preferences [10]. Moreover, end-of-life care for older adults and people with severe disabilities is commonly provided in long-term care or disability welfare facilities rather than hospices [2,10]. Family centered decision-making and reluctance to discuss prognosis directly further influence care planning [10]. These structural and cultural characteristics further intensify the challenges of caring for patients with SMID, whose inherent communication barriers already limit the feasibility of shared decision-making.

Culturally meaningful psychosocial interventions, including music therapy, have been shown to reduce anxiety, improve mood, and enhance the quality of life in terminally ill patients [11,12]. Such interventions may be particularly valuable for patients with profound cognitive impairment, for whom nonverbal sensory-based modalities can serve as important channels for emotional expression and comfort. In this context, patient responses to psychosocial interventions were evaluated primarily through qualitative clinical observations by family members and the care team. To provide a limited, purely illustrative visualization of the qualitatively observed increase in smiling behavior after music therapy, we conducted a supplementary, non‑quantitative, and exploratory review using OpenFace [13].

This case report describes the end-of-life care of an elderly man with SMID and peritoneal carcinomatosis of unknown primary origin. By integrating surrogate-led decision-making, multidisciplinary collaboration, continuity of setting, and a personalized live music intervention, this case illustrates how culturally tailored, preference-based palliative care can support comfort, engagement, and dignity at the end of life for patients who cannot advocate for themselves.

## Case presentation

A 68-year-old man with SMID and cerebral palsy due to hypoxic-ischemic encephalopathy from perinatal asphyxia had been residing in a long-term care facility for 58 years. He required full assistance with activities of daily living and had no functional verbal communication; however, he consistently demonstrated emotional responses to preferred stimuli such as music and specific foods. His medical history was notable for chronic dermatological issues, including recurrent erythema, urticarial wheals, and excoriation of the abdomen, thighs, forearms, back, and buttocks. He also experienced seasonal infectious episodes such as influenza, purulent nasal discharge, and prolonged cough, as well as minor trauma associated with daily activities, including digital bleeding and mild head injuries. According to staff reports, these head injuries were mild and occurred during routine daily activities; however, detailed documentation regarding their specific types or causes was not available. Regarding preventive immunization, the patient did not receive a pneumococcal vaccination, but an annual influenza vaccination was administered. Before being diagnosed with advanced cancer, the patient was taking routine oral medications, including cetirizine, alpha-calcidol, and amlodipine, without difficulty. Around day 102, he was also administered oral sitafloxacin for a urinary tract infection. No intravenous medication was administered.

Over the preceding two years, the patient had experienced gradual but clinically significant weight loss from 48.1 kg to 41.7 kg (a total loss of 6.4 kg). Because the patient was nonverbal and unable to report symptoms, this subacute-to-chronic decline raised concerns about an underlying systemic or malignant process. Since the patient was residing in the facility for an extended period, for this report, day one is defined as the day when thoracic and abdominal computed tomography (CT) scans were performed to investigate the cause of his weight loss, to evaluate occult infection, malignancy, or other intrathoracic or intra-abdominal pathologies. CT revealed multiple pulmonary metastatic nodules, a nodular lesion in the spleen, and multiple peritoneal implants (Figures 1A, 1D, 1G).

**Figure 1:**
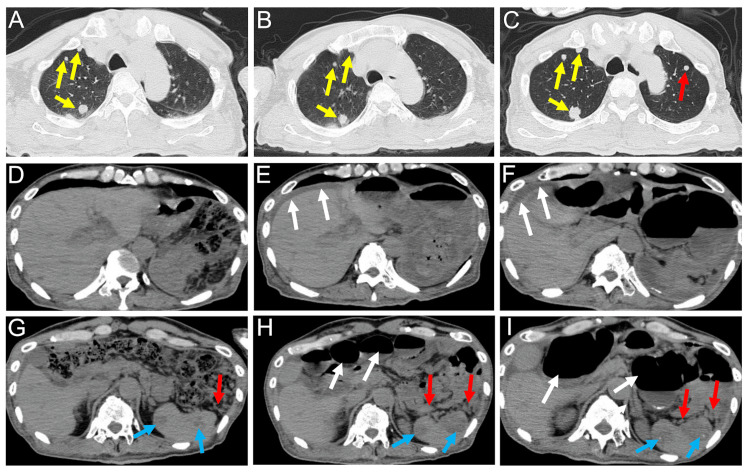
Thoracic and abdominal CT findings over time A–C: Thoracic CT scans obtained on days 1, 83, and 119, respectively. Multiple metastatic nodules are observed in the right upper lung field (yellow arrows in A–C). On day 119, a new metastatic nodule is identified in the left upper lung field (red arrow in C). D–F: Upper abdominal CT scans on days 1, 83, and 119, respectively. Perihepatic ascites was present on days 83 and 119 (white arrows in E and F). G–I: Upper abdominal CT scans on days 1, 83, and 119, respectively. Splenic nodular lesions are visible on day 1 (blue arrows in G). On days 83 and 119, the size of the splenic nodular lesions increased (blue arrows in H and I). Multiple peritoneal implants suggestive of metastatic peritoneal dissemination were also observed (red arrows in G–I). Intestinal dilation is evident on days 83 and 119 (white arrows in H and I, respectively).

Laboratory tests performed on day five revealed markedly elevated levels of tumor markers, including carcinoembryonic antigen (CEA) at 18.4 ng/mL and carbohydrate antigen 19-9 (CA19-9) at 544 U/mL (Table 1).

**Table 1:** Changes in laboratory findings from illness onset Marked elevations in tumor marker levels, including CEA and CA19‑9, were observed on day 5. The CRP levels increased on day 102. An elevation in the hemoglobin level was observed on day 118, consistent with hemoconcentration. WBC, white blood cell; Hb, hemoglobin; CRP, C-reactive protein; CEA, carcinoembryonic antigen; CA19-9: carbohydrate antigen 19‑9; N/A, not available

	Day 5	Day 102	Day 118	Reference value
WBC (/mL)	5300	8200	15000	3900-9800
Hb (g/dL)	14.3	14.4	18.4	13.5-17.6
CRP (mg/dL)	1.1	18.1	3.6	<0.30
CEA (ng/mL)	18.4	N/A	N/A	<5.0
CA19-9 (U/mL)	544	N/A	N/A	<37

The patient was referred to a university hospital on day 16 when he was diagnosed with stage IV CUP. Based on the abdominal ultrasound findings, hepatocellular carcinoma, gallbladder carcinoma, pancreatic carcinoma, renal carcinoma, and bladder carcinoma were considered unlikely. The estimated prognosis was three to 10 months.

Owing to impaired decision-making capacity, the patient’s legal guardian, his aunt, served as the surrogate decision-maker. She declined invasive diagnostic procedures, chemotherapy, and radiotherapy, prioritizing comfort-focused care. Therefore, further investigations to identify the primary tumor site were not pursued. Given the patient’s frailty, severe comorbidities, and the limited clinical benefits expected from additional invasive procedures, further diagnostic workup was deemed inappropriate. His legal guardian strongly preferred that the patient remain in the familiar environment of the facility to avoid distress associated with transfer. A Do Not Attempt Resuscitation (DNAR) order was proposed by his legal guardian on day five. The overarching goals of care are to minimize physical burden, maintain comfort and dignity, and preserve familiar routines and preferred activities. Based on the “Omoi wo Tsunagu Note,” which is the advance care planning (ACP) tool used at our center in accordance with the guidelines issued by the Ministry of Health, Labour and Welfare of Japan, we examined the type of care that would be desirable for the patient through multidisciplinary collaboration on day 21 [10]. Based on the results of this review, we discussed appropriate care and treatment options with the patient’s legal guardian on day 33, during which a DNAR order was formally established. During the same discussion, she proposed planning a memorial concert to be held within a long-term care facility, as this was considered to reflect the patient’s wishes.

For approximately one month after diagnosis, the patient participated in wheelchair outings, including shopping and attending facility events. At the events, he enjoyed preferred foods such as cheesecake and takoyaki (traditional Japanese cuisine). He routinely enjoyed listening to nursery songs on the CD and engaged in active music‑making, striking piano keys, and producing singing-like vocalizations, although without a recognizable melody. Snoezelen-based relaxation sessions were also been conducted.

On day 47, the patient was informed of the formal involvement of the palliative care team. During this discussion, we reaffirmed that medical interventions would be limited to the minimum necessary to alleviate discomfort. On day 54, as part of the MDT’s ongoing ACP efforts, we discussed preparations for organizing the memorial concert, ways to incorporate the patient’s preferred foods, and plans for outdoor walks. During a follow-up discussion on day 70, it was decided that his favorite foods, takoyaki three times per week and cheesecake once per week, would be provided on a regular basis.

On day 83, the patient experienced recurrent vomiting of food residue and watery diarrhea. CT revealed intestinal dilatation, increased ascites, and abdominal nodules suggestive of peritoneal metastases (Figures 1B, 1E, 1H). Because he showed signs of impending ileus, oral intake was temporarily discontinued and he was limited to minimal amounts of water. We explained that as dehydration were to progress, minimal intravenous fluids should be considered, and obtained consent for this plan from his legal guardian. After the vomiting and diarrhea resolved, his diet was gradually modified from soft rice to blenderized meals, and eventually to minimal oral intake. During a palliative care conference involving the MDT on day 95, we discussed the potential use of corticosteroids, possibility of draining ascites, and need for intravenous fluids. Ultimately, it was agreed that the overall goal of care would be to prioritize patient comfort and align all interventions with the intentions of the patient’s legal guardian. On day 99, during a discussion with his legal guardian, she expressed her wishes that intravenous fluids be limited to the minimum necessary dose, corticosteroids not be used, and no drainage of ascites be performed.

On day 102, he developed a urinary tract infection characterized by pink-tinged urine, fever of 38.0°C, and elevated CRP at 18.1 mg/dL (Table 1). The patient was treated with oral sitafloxacin, and his fever subsided temporarily. By day 119, his oral intake had declined to only a few spoonfuls of ice cream and occasional sips of water. CT revealed a new metastatic nodule in the left upper lung field and worsening bowel dilatation (Figures 1C, 1F, 1I). Intravenous fluids were minimized due to concerns about edema and in accordance with the preferences of the patient’s legal guardian. During a discussion with his legal guardian, it was reaffirmed that minimal intravenous hydration would be acceptable if needed to help maintain his condition for participation in the memorial concert scheduled four days later; however, no intravenous fluids were ultimately administered.

Given the patient’s lifelong affinity for music, his legal guardian organized a personalized end-of-life cultural intervention. On day 123, a personalized “Memory Concert” featuring piano, violin, and flute performances of nursery songs and familiar Studio Ghibli melodies was held. The patient’s family, including his legal guardian, reported that his facial expressions became noticeably more relaxed during the music session, with more smiles and visible reactions, and that he remained calm afterward. They also noted that the gathering of relatives, some of whom lived far away, who came together in person for the “Memory Concert” created an opportunity for the family to reconnect around the patient, which, in their view, deepened his sense of comfort and emotional connectedness. To offer a limited visual illustration of these family reported impressions rather than to generate any formal quantitative evaluation, we conducted a simple referential comparison of two facial photographs, one obtained approximately five months prior and one captured during the music therapy session, using OpenFace (version 2.0), an open-source toolkit grounded in the Facial Action Coding System (FACS) [13]. The comparison focused on AU6_r and AU12_r, which characterize the Duchenne smile. This exploratory use of FACS-based metrics was intended solely as a descriptive adjunct to the family’s observations, and not as a scientific or quantitative assessment. Within this limited illustrative context, the findings appeared broadly consistent with the family’s impression of increased positive facial expressions during music therapy session (Table 2).

**Table 2:** Illustrative comparison of facial action units before and during the music therapy session This table presents a descriptive, non-quantitative comparison of automated facial action unit (AU) estimates derived from two static photographs. Small increases in AU6_r (cheek raiser) and AU12_r (lip corner puller) were observed in images captured during the music session. In OpenFace, the “_r” suffix denotes regression-based AU intensity estimates (range 0–5). These values are presented solely as illustrative adjuncts to family reported impressions and should not be interpreted as quantitative evidence of emotional states or therapeutic effects.

	Before music therapy session	During music therapy session
AU6_r (cheek raiser)	0.6	1.52
AU12_r (lip corner puller)	0.44	1.98

On the days following the concert, a temporary increase in fluid intake was observed; however, this association did not establish causality (Figure 2).

**Figure 2:**
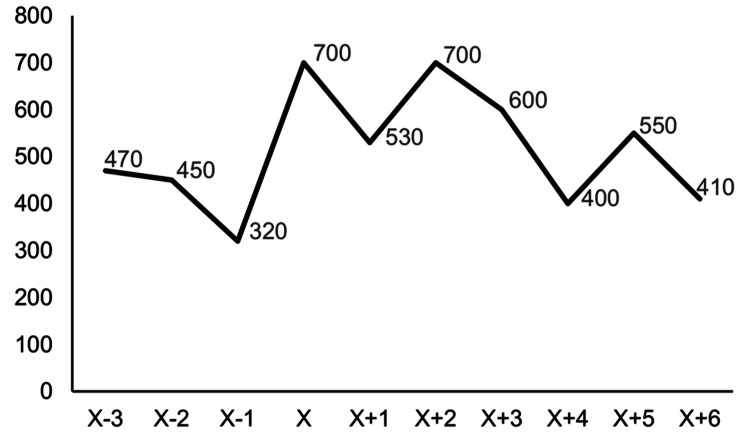
Changes in daily oral fluid intake before and after the music therapy session Daily oral fluid intake (mL) is plotted with the day of the music therapy session designated as X. Values from three days before (X−3) to six days after (X+6) are shown. Fluid intake increased immediately after the session and remained higher than the pre intervention period until X+3, followed by a gradual decline after X+4.

He was observed to be more alert and engaged than in the previous days. The patient’s legal guardian reported that the patient’s spontaneous fluid intake after the session was particularly meaningful and encouraging. Six days after the concert, his condition began to decline again, and he developed tachypnea and decreased responsiveness. On the morning of the seventh day after the concert, he exhibited shallow breathing, peripheral cooling, and mottled cyanosis, and his blood pressure was unmeasurable. During his final hours on the seventh day after the concert, his legal guardian remained at the bedside, holding his hand and providing comfort. The patient died early in the evening following cessation of respiration and cardiac activity. No signs of distress were observed at death. The patient’s family, including his legal guardian, expressed gratitude that the intervention contributed to what they perceived as a peaceful and desirable end-of-life experience without signs of distress.

A memorial conference held one month after death concluded that the patient experienced a dignified and comfortable death aligned with the values of his legal guardian and the goals of ACP.

Ethical considerations

1) Patient capacity: The patient lacked decision-making capacity at the time of care; 2) Consent for music therapy: Consent for participation in music therapy was obtained in accordance with institutional policy; 3) Identifiable information: No identifiable images or videos are included in this case report; 4) Next‑of‑kin permission: No media materials were used without explicit next‑of‑kin permission; 5) IRB approval: This study was approved by the Medical Research Ethics Committee of Nishinomiya Sunago Medical and Welfare Center; 6) Written informed consent for publication was obtained from the patient’s legal guardians; 7) Data availability: All relevant data are contained within the manuscript. 

## Discussion

This case highlights several important clinical insights regarding end-of-life care for patients with SMID who develop advanced malignancies such as CUP. First, the single-session live music intervention generated visible joy and emotional engagement, demonstrating the therapeutic potential of culturally meaningful sensory interventions. Second, culturally tailored, preference-based care contributes to peaceful and dignified end-of-life experiences. These observations underscore the importance of adopting a holistic, person-centered approach that integrates the medical, ethical, and cultural dimensions of care.

CUP presents substantial diagnostic and therapeutic challenges due to its heterogeneous nature and advanced stages, which are typically clinically apparent [7-9]. In this case, the patient’s intellectual disability further limited the feasibility of invasive diagnostic procedures and precluded conventional shared decision-making. Individuals with intellectual disabilities often experience heightened vulnerabilities during hospitalization, including anxiety, sensory overload, and difficulty adapting to unfamiliar environments [3-5]. For this population, maintaining a continuous setting, such as remaining in a long-term care facility, can be essential for minimizing distress and preserving a sense of security [3-6]. In this case, the decision to avoid hospital transfer and continue care within the familiar environment of a long-term care facility played a central role in supporting patient comfort.

The involvement of MDT is also critical. Clinicians, nurses, caregivers, and facility staff collaborated to interpret patients’ behavioral cues, assess comfort, and coordinate interventions aligned with their established routines and psychosocial needs. This collaborative framework allows the team to integrate medical management with culturally grounded practices, ensuring that care remains consistent with the patient’s identity and lived experiences.

Cultural and psychosocial interventions have become increasingly recognized as essential components of holistic palliative care. Music therapy has been shown to reduce anxiety, improve mood, and enhance the quality of life of terminally ill patients [11,12]. The “Memory Concert” in this case served not only as a personalized cultural intervention but also as a means of honoring the patient’s long-standing preferences and life history. The patient’s visible emotional response, smiling, vocalizing, and engaging with music, suggests that culturally meaningful sensory experiences can provide comfort and connections even in the context of profound cognitive impairment.

In the context of end-of-life care for individuals with SMID, profound communication barriers and autonomic instability often limit the interpretability of standard assessment scales and physiological indicators [14,15]. Previous literature has emphasized the importance of sustained multidisciplinary clinical observation as a reliable means of assessing comfort in this population [14,15]. Although music therapy is beneficial in end-of-life settings, reports involving individuals with SMID are scarce. In this case, the patient’s discomfort appeared to lessen without the use of opioids, suggesting that qualitative non-pharmacological approaches may represent a potentially meaningful option for this group.

ACP is indispensable to individuals with impaired decision-making capacity [16,17]. Surrogate decision-making requires careful ethical deliberation, particularly when patients cannot express their preferences verbally. In this case, the legal guardian’s decisions were informed by years of familiarity with the patient’s habits, preferences, and behavioral expressions. The MDT supported this process by providing the clinical context and ensuring that care decisions were aligned with medical realities and patient values. This ethically grounded, surrogate-led approach contributed to peaceful and dignified deaths, demonstrating how individualized, culturally responsive palliative care can be effectively implemented, even in highly complex clinical situations (Figure 3).

**Figure 3:**
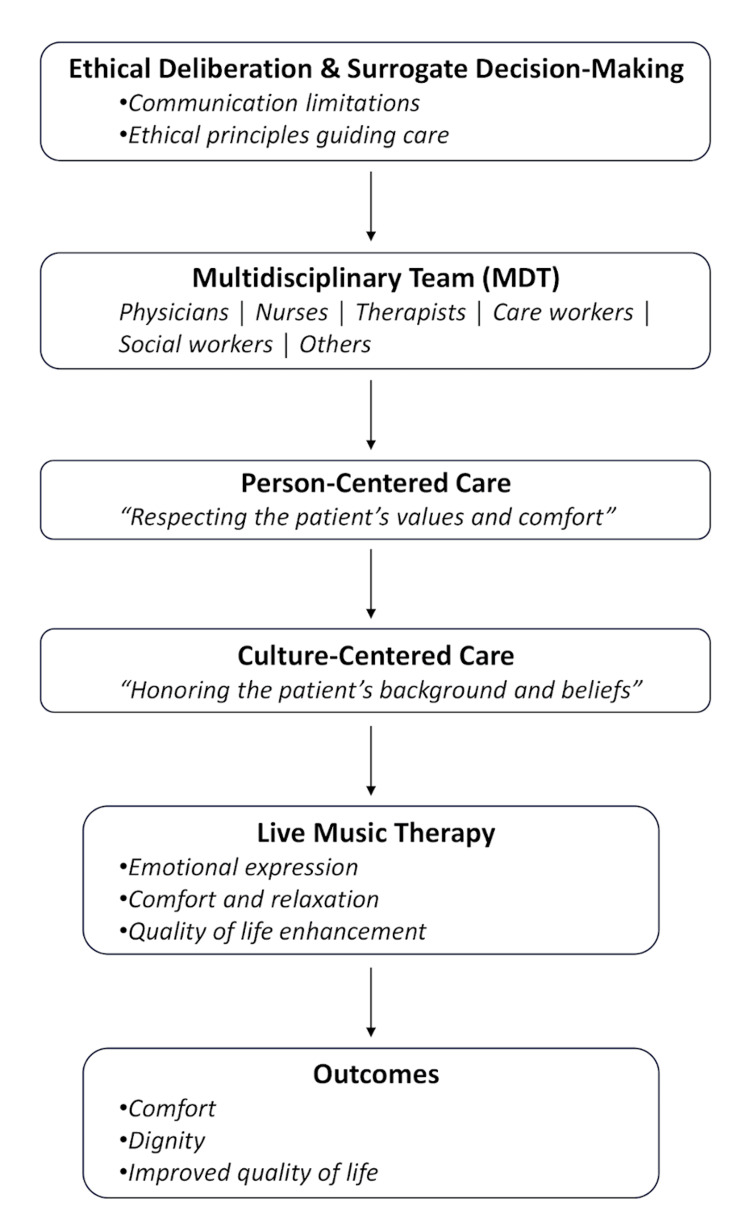
Conceptual framework for an end-of-life care approach for older patients with severe motor and intellectual disabilities and cancer of unknown primary This conceptual framework illustrates the end-of-life care approach adopted in our facility for older patients with severe motor and intellectual disabilities (SMID) complicated by cancer of unknown primary (CUP). Because verbal and linguistic communication is often difficult in this population, care is guided by ethical considerations and surrogate decision-making supported by a multidisciplinary team (MDT). This approach emphasizes person-centered and culture-centered care, ensuring that the patient’s values, comfort, and lived experiences remain central throughout the decision-making process. In the present case, live music therapy was incorporated as a non-pharmacological intervention to promote emotional expression and enhance the quality of life.

In surrogate decision-making for individuals with SMID, an important ethical distinction exists between the substituted judgment standard and the best interest standard. Substituted judgment relies on inferring what the patient would have chosen based on previously expressed preferences or established values [18]. However, in cases such as this one, where the patient has never possessed decision-making capacity, a strict substituted judgment is not applicable [18]. Therefore, decisions must rely primarily on the best-interest standard, which emphasizes the relief of suffering, preservation of dignity, and overall welfare. At the same time, the best interest standard has been criticized for the risk of reflecting the values of caregivers rather than those of patients [19]. To mitigate this concern, the MDT and legal guardian incorporated long-term observations of the patient’s behavioral patterns, comfort cues, and culturally meaningful preferences. Although these do not constitute autonomous choices, integrating them into the decision-making process helps align best-interest assessments with the patient’s lived identity, thereby strengthening the ethical justification for surrogate-led end-of-life care for individuals with lifelong cognitive impairment [19].

To contextualize these findings, it is important to acknowledge the inherent limitations of this single case report. First, invasive diagnostic procedures could not be performed, restricting the precision of the clinical assessment. Second, evaluating the impact of cultural interventions using a single-case design presents inherent methodological challenges, and the findings should not be interpreted as evidence of causal relationships. Although FACS is optimized for dynamic video analysis, our use of static-image AU estimation lacked temporal information and was intended solely as an illustrative, non-quantitative adjunct to family reported observations. No formal assessment of consciousness such as the Standardized Canadian Neurological Scale (CNS), was conducted during the observation period. We also acknowledge that in addition to fluctuations in the patient’s level of consciousness, a recent urinary tract infection may have influenced oral intake, which represents an important limitation when interpreting the observed changes. Finally, the conclusions of this case report rely primarily on qualitative observations, including clinical impressions of the care team, qualitative behavioral changes, and the temporal context of the music therapy session, rather than on quantitative measures of causality.

Taken together, this case illustrates the importance of integrating ethical decision-making, multidisciplinary collaboration, continuity of setting, and culturally meaningful interventions when providing end-of-life care for patients with SMID and advanced cancer. These elements collectively support a model of care that is not only medically appropriate but also deeply respectful of the patient’s identity, preferences, and dignity.

## Conclusions

This case illustrates that end-of-life care for patients with SMID and advanced cancer requires an ethically grounded multidisciplinary approach that integrates surrogate decision-making, continuity of settings, and culturally meaningful interventions. The patient’s legal guardian played a central role in ensuring that care decisions reflected his long-standing preferences and minimized distress, whereas the MDT supported the delivery of person-centered care tailored to his cognitive and functional needs. The incorporation of a personalized music-based intervention - the “Memory Concert” - demonstrated the therapeutic value of culturally rooted practices in promoting comfort, emotional engagement, and dignity at the end of life. Clinicians should remain attentive to the unique vulnerabilities of patients with SMID, prioritize familiar environments, and collaborate closely with surrogate decision-makers to deliver individualized, compassionate, and culturally responsive palliative care.
